# Supplementary material for: Development of chemokine network inhibitors using combinatorial saturation mutagenesis
Source: Commun Biol. 2025 Apr 3;8:549. doi: 10.1038/s42003-025-07778-6 (PMC11969024; doi:10.1038/s42003-025-07778-6)
Supplement: Supplementary file 4 — Supplementary Data 2 [file 42003_2025_7778_MOESM4_ESM.zip › SupplementaryData3/scripts/xgeo.tar!/./script/analysis/JK/GSE198520_homo_rheumatoid_synovium_boxplot.pdf]

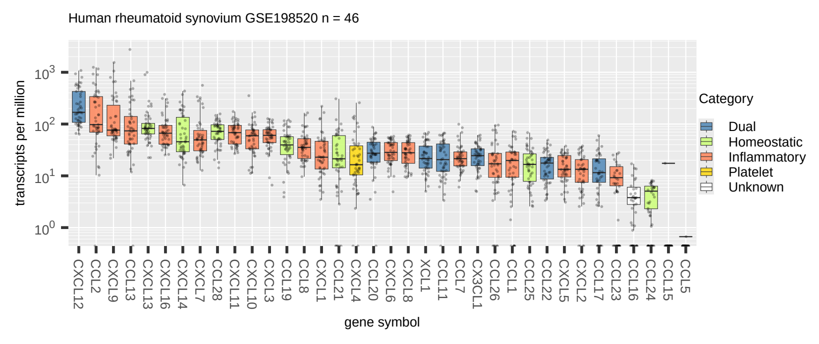

Human rheumatoid synovium GSE198520 n = 46
transcripts per million
10^3
10^2
10^1
10^0
CXCL12
CCL2
CXCL9
CCL13
CXCL13
CXCL16
CXCL14
CXCL7
CCL28
CXCL11
CXCL10
CXCL3
CCL19
CCL8
CXCL1
CCL21
CXCL4
CCL20
CXCL6
CXCL8
XCL1
CCL11
CCL7
CX3CL1
CCL26
CCL1
CCL25
CCL22
CXCL5
CXCL2
CCL17
CCL23
CCL16
CCL24
CCL15
CCL5
gene symbol
Category
Dual
Homeostatic
Inflammatory
Platelet
Unknown
